# Real-Time Occlusion Handling in Augmented Reality Based on an Object Tracking Approach

**DOI:** 10.3390/s100402885

**Published:** 2010-03-29

**Authors:** Yuan Tian, Tao Guan, Cheng Wang

**Affiliations:** Digital Engineering & Simulation Research Center, Huazhong University of Science and Technology, Wuhan 430074, China; E-Mails: tianyuancolor@gmail.com (Y.T.); wangch@hhu.edu.cn (C.W.)

**Keywords:** augmented reality, occlusion, tracking, mean shift, optical flow, graph cuts

## Abstract

To produce a realistic augmentation in Augmented Reality, the correct relative positions of real objects and virtual objects are very important. In this paper, we propose a novel real-time occlusion handling method based on an object tracking approach. Our method is divided into three steps: selection of the occluding object, object tracking and occlusion handling. The user selects the occluding object using an interactive segmentation method. The contour of the selected object is then tracked in the subsequent frames in real-time. In the occlusion handling step, all the pixels on the tracked object are redrawn on the unprocessed augmented image to produce a new synthesized image in which the relative position between the real and virtual object is correct. The proposed method has several advantages. First, it is robust and stable, since it remains effective when the camera is moved through large changes of viewing angles and volumes or when the object and the background have similar colors. Second, it is fast, since the real object can be tracked in real-time. Last, a smoothing technique provides seamless merging between the augmented and virtual object. Several experiments are provided to validate the performance of the proposed method.

## 1. Introduction

The objective of augmented reality (AR) is to superimpose computer enhancements on the real world. In contrast with virtual reality, users can see virtual objects and the real world simultaneously in the augmented reality system. The applications of augmented reality are extensive: computer-aided surgery [1], entertainment [2], education [3], tourism industry [4], military exercises [5], and product design [6].

**Figure 1.**
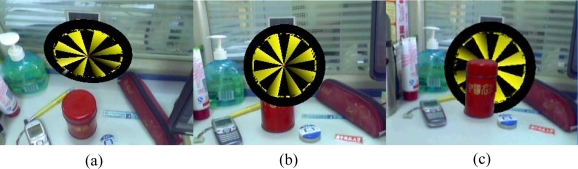
Occlusion problem in augmented reality.

Until now, considerable research has been done on the head-mounted display and the registration problem, which deals with the consistency between the coordinate systems of the real and virtual worlds [7–11]. To enhance the illusion that the virtual objects are actually present in the real scene, researchers have paid more and more attention to the occlusion problem. The problem of occlusion occurs when the real objects are in front of the virtual objects in the scene. Without occlusion handling, users will have the misconception that the real object is further from the viewpoint than the virtual objects when the virtual objects are occluded by the real objects in the scene. This not only leads to misconceptions of spatial properties by the user, resulting in errors when trying to grab objects, but also increases eyestrain and the probability of motion sickness [12]. An example of an occlusion problem is shown in Figure 1. In Figure 1, the virtual object is a target. Figure 1a is the composed image looking down from the top of the scene. We can find that the target is behind the red caddy if we see from the viewpoint as shown in Figure 1c. This means that part of the target should be occluded by the red caddy. Figure 1b is the composed image overlaid with virtual target without occlusion handling. The image gives people the impression that the target is in front of the caddy, which is not the case in fact.

## 2. Related Work and Our Contribution

Various approaches have been suggested for handling occlusion problems in augmented reality. They are mainly classified into two types: model-based and depth-based approaches.

The precondition of utilizing a model-based approach is that accurate geometric models of real objects must be known. Fuhrmann *et al.* [12] simulate the occlusion of virtual objects by a representation of the user modeled as kinematic chains of articulated solids. This method is limited to static scenes. Ong *et al.* [13] used the user-segmented object silhouettes in the key frames to build the three-dimensional (3D) model of the occluding object. The 2D occluding boundary is then obtained by projecting the 3D shape in the intermediate frames. However, along with the motion of the viewpoint, the 3D model will change. The projection of the recovered 3D shape cannot precisely reflect the 2D occluding boundary in the intermediate frames. Vincent *et al.* [14] added two improvements: First, they computed the 3D occluding boundary from two consecutive key views instead of all the key views. Second, the accurate occluding boundary was recovered using a region-based tracking method. In addition, they refine the occluding boundary based on snakes. The drawback of this approach is that it can not deal with large viewpoint variations.

Berger [15] presented a new approach for resolving occlusions without 3D reconstruction. The main idea is to label each contour point of the real object as being “behind” or “in front of”, depending on whether it is in front of or behind the virtual object. Schmidt *et al.* [16] used a binocular stereo camera system to obtain proper occlusions. The disparity calculation method is improved and extremely efficient. Hayashi *et al.* [17] proposed a method for real-time stereo matching using a contour based approach to acquire the accurate depth of the boundary of real objects. Fortin *et al.* [18] described how to extract a depth map of the scene along the viewpoint. Then the depth buffer is used to handle occlusions.

The model-based and depth-based approaches have their own disadvantages. In the model-based method, the accuracy of 3D reconstruction has a great influence on the results of occlusion handling. However, the contour of the real object will change when the viewpoint of the camera changes. This will lead to reconstruction errors and inaccuracies in the 2D occluding boundary. Furthermore, the 3D models of the real object need to be reconstructed off-line, so the model-based method is not suitable for real-time occlusion handling. The depth-based approach is based on stereo vision theory. The disadvantages are as follows: (1) the expensive computation time and inaccurate depth information; (2) the complex calibration process of the stereo cameras; 3) the narrow application range, since this is only suitable for static real scenes.

In this paper, we develop a new approach for effectively handling occlusion in real-time. Our method is different from the model-based and depth-based methods mentioned above. The proposed method distinguishes itself in the following ways:

First, we use an improved interactive object segmentation method based on mean shift and graph cuts to obtain the contour of the specified occluding object in the first frame. The proposed segmentation method finds the object boundary even though the scene is complex.

Second, a real-time method combining graph cuts and optical flow is used to track the object in the subsequent frames. This method tracks objects robustly, even when the object contains many colors that are similar as its background or the camera is moved through large changes of viewing angles and volumes. Moreover, it has the ability to converge onto the object boundary within a few frames, even when the previous object boundary is not properly initialized.

Third, we propose a way to obtain correct occlusion relationships by redrawing all the pixels of the tracked object on the augmented image. Furthermore, the boundary between the occluding and physical object is made seamless by a smoothing process.

The remaining parts of this paper are organized as follows. Section 3 gives an overview of the proposed approach, while Section 4, Section 5 and Section 6 present in detail the initial selection of the occluding object, the tracking method and occlusion handling approach, respectively. Section 7 shows the experimental results. Finally, conclusions and future work are given in the last section.

## 3. Overview of the Proposed Approach

**Figure 2.**
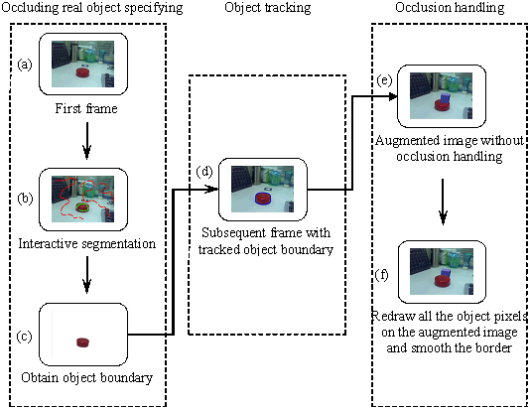
Work Flow of the Proposed Approach.

In the augmented reality systems, virtual objects are usually rendered on the video image without using depth information from the real scene, so real objects are always occluded by virtual objects [17]. We call this the “occlusion problem”, as shown in Figure 1. This problem results in poor understanding of the geometrical relationship between real and virtual objects. The goal of our method is to obtain the correct relative position when the real object is in front of the virtual object. The work flow of the proposed approach is shown in Figure 2.

Our method consists of three steps:

1. Select the real object that will occlude the virtual object in the first frame. This selection process is accomplished by an interactive interface, where the user labels some pixels as foreground and others as background. All the pixels in the image are divided into two classes, object and background, according to the hidden information provided by the labeled pixels. Finally, the object boundary is obtained.
2. Track the object boundary in the subsequent frames. This is done by extracting feature points on the object in the previous frame and tracking these points in the current frame. The object boundary is estimated according to the average displacement of the tracked feature points. Then the accurate object boundary is found in the banded area that is around the estimated boundary.
3. Redraw all the pixels inside the object boundary on the augmented image to correct the relative position between the real and virtual object. A smoothing operation is utilized on the object boundary to make a more realistic augmentation.

We will describe our approach in more detail in the following sections.

## 4. Selecting the Occluding Object

The physical occluding object is specified in the first frame by an interactive image segmentation method. This section describes our interactive image segmentation method, which is based on the mean shift algorithm and graph cuts techniques [19]. The user selects several pixels by marking lines to specify the physical occluding object and a few pixels on the background as shown in Figure 2b. This divides the pixels in the image into three types: foreground pixels {*F*}, background pixels {*B*} and unlabeled pixels {*U*}. The final goal is to assign each unlabeled pixel as either foreground or background. Our method includes three steps: color space selection, labeled pixel analysis based on mean shift and object extraction using a graph-based method. The steps are as follows:

### 4.1. Color Space Selection

The selection of color space is important for the performance of image segmentation. It is proven that the LUV color space is superior to the RGB color space in image processing. In LUV color space, L^*^ represents the lightness and U^*^ and V^*^ denote the chromatism. The distances between different colors are defined as:

(1) $$\Delta C=\sqrt{{\left(L_{a}^{*}-L_{b}^{*}\right)}^{2}+{\left(U_{a}^{*}-U_{b}^{*}\right)}^{2}+{\left(V_{a}^{*}-V_{b}^{*}\right)}^{2}}$$

From the above definition, it is clear that the closer the two pixels are in LUV color space, the smaller the color difference. Therefore, the Euclidean metrics and distances are perceptually uniform in LUV color space.

### 4.2. Labeled Pixels Analysis Based on Mean Shift

The manual user interaction provides clues as to which object in the image he intends to segment. An analysis of the properties of the foreground and background pixels selected by the user is done by applying the mean shift algorithm [20]. The mean shift method is a simple iterative procedure that shifts each data point to the average of the data points in its neighborhood [21]. For a color image, the image is presented as a vector *x* = (*x^s^*, *x^r^*) of five dimensions, where *x^s^* is the spatial part and *x^r^* is the range part of the feature vector. Given *n* image pixels *x_i_*, *i* = 1,…, *n* in the *d* -dimensional space *R^d^* (*d* = 5 for color images), the radially symmetric kernels, which we use satisfy

(2) $$K(x)=c_{k,d}k({\left\|x\right\|}^{2})$$

where the function *k*(*x*) is referred to as the profile of the kernel, but only for *x* ≥ 0. The normalization constant *c_k,d_*, which makes *K*(*x*) integrate to one, is assumed to be strictly positive. Define *g*(*x*) = −*k*′(*x*) for profile, the kernel *G*(*x*) is defined as

(3) $$G(x)=c_{g,d}g({\left\|x\right\|}^{2})$$

where *c_g,d_* is the corresponding normalization constant. The MS is defined as

(4) $$m_{h,G}(x)=\frac{\sum_{i=1}^{n}x_{i}g({\left\|\frac{x-x_{i}}{h}\right\|}^{2})}{\sum_{i=1}^{n}g({\left\|\frac{x-x_{i}}{h}\right\|}^{2})}-x$$

where *x* is the center of the kernel, and *h* is the bandwidth parameter satisfying *h* > 0. Therefore, the MS is the difference between the weighted mean and *x*. The weighted mean uses the kernel *G* as the weights. The center position of kernel *G* can be updated iteratively by

(5) $$y_{i+1}=\frac{\sum_{i=1}^{n}x_{i}g({\left\|\frac{y_{j}-x_{i}}{h}\right\|}^{2})}{\sum_{i=1}^{n}g({\left\|\frac{y_{j}-x_{i}}{h}\right\|}^{2})},\;\;\;\;\;\;\;\;\;j=1,2,\ldots$$

where *y*_1_ is the center of the initial position of the kernel. The center of each cluster is obtained when Equation (5) converges. This divides the labeled pixels into *n* regions denoted as { $R_{n}^{F}$} for the foreground and *m* regions denoted as { $R_{m}^{B}$} for the background. { $R_{n}^{F}$} and { $R_{m}^{B}$} are two sets of mean pixel intensities of the clustered regions in the three different color spaces.

### 4.3. Object Extraction Using a Graph-Based Method

The next step is to segment the image using the approach described in [22], where a specialized graph that reflects the properties of the image is constructed. In the graph, each node denotes a pixel in the image and each edge connecting two adjacent nodes is assigned a nonnegative weight. Two special nodes, called foreground terminal (a source) and background terminal (a sink), are defined. So there are three types of edges: *e*(*p*, *q*) (pixel-pixel) with edge weight *w_e_*_(_*_p,q_*_)_, *e*(*p*, *S*) (pixel-source) with edge weight *w_e_*_(_*_p,S_*_)_ and *e*(*p*, *T*) (pixel-sink) with edge weight *w_e_*_(_*_p,T_*_)_. The edge weights are defined as follows:

(6) $$w_{e(p,q)}=\exp(-\frac{{\left(I_{p}-I_{q}\right)}^{2}}{{2\sigma }^{2}})\cdot \frac{1}{\mathrm{dist}(p,q)}$$

where *dis*(*p*, *q*) is the spatial distance from *p* to the neighborhood pixel *q*.

(7) $$w_{e(p,S)}=\{\begin{array}{ccc} \frac{d_{i}^{B}}{d_{i}^{F}+d_{i}^{B}} & \mathrm{if} & p\in U\\ \infty & \mathrm{if} & p\in F\\ 0 & \mathrm{if} & p\in B \end{array}\;\;\;\;\;\;\;\;\;w_{e(p,T)}=\{\begin{array}{ccc} \frac{d_{i}^{F}}{d_{i}^{F}+d_{i}^{B}} & \mathrm{if} & p\in U\\ 0 & \mathrm{if} & p\in F\\ \infty & \mathrm{if} & p\in B \end{array}$$

where $d_{i}^{F}$ is the minimum distance from the color *I_p_* of pixel *p* to foreground clusters { $R_{n}^{F}$}, and similarly, $d_{i}^{B}$ is the minimum distance from its color *I_p_* to background clusters { $R_{m}^{B}$}.

For a pixel-pixel edge, the more similar the two adjacent pixels are, the larger the weight is, and thus the two adjacent pixels are more likely to be assigned to the same class (foreground or background). The edge weights of pixel-source and pixel-sink reflect the penalties that assign pixels to foreground and background. They may reflect how the pixel fits into the classification provided by the user interaction. For a labeled foreground pixel, the user has specified that it belongs to the foreground and it can never be assigned as a background pixel, so the pixel-source edge weight is set to infinite and the pixel-sink edge weight is set to zero. Similarly, for a labeled background pixel, the pixel-sink edge weight is set to zero and the pixel-source edge weight is set to infinite. For an unlabeled pixel, the edge weight of pixels-source will be large when the pixel has a similar color to the foreground clusters, thus it is more likely to be assigned to the foreground. By contrary, the edge weight of pixels-sink will be large when the pixel has similar color to the background clusters, thus it is more likely to be assigned to the background.

After constructing the graph and setting all the edge weights in the graph, we use the maximum flow-minimum cut algorithm described in [23] to find the cut that minimizes the sum of the edge weights:

(8) $$\mathrm{Cut}=\min(\lambda \cdot \sum w_{e(p,q)}+\sum w_{e(p,S)}+\sum w_{e(p,T)})$$

where the coefficient *λ* ≥ 0 specifies a relative importance of *w_e_*_(_*_p,q_*_)_ versus *w_e_*_(_*_p,S_*_)_ and *w_e_*_(_*_p,T_*_)_. The result is that each pixel in the image is assigned to the foreground or background exclusively as shown in Figure 2c.

## 5. Occluding Real Object Tracking

**Figure 3.**
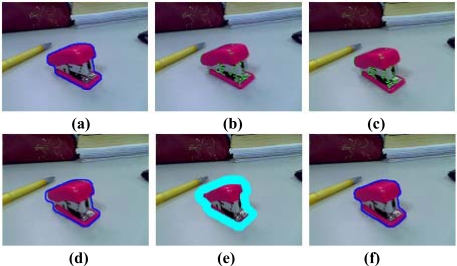
The steps of object tracking. (a) The object contour C*_t_*_−1_ at time t − 1. (b) The features detected in frame *I* at time *t* − 1. (c) The tracked features in frame *J* at time *t*. (d) The coarse contour *Ĉ_t_* of frame *J* at time *t*. (e) The banded area *B_t_*. (f) The accurate contour C*_t_* of frame *J* at time *t*.

To make the relationship between the physical and virtual object correct, the contour of the physical object should be tracked in subsequent frames. In this paper, the object contour is tracked using a graph cuts based method motivated by Xu *et al.* [24] and Lombaert *et al.* [25]. The steps are illustrated in Figure 3. Given the object contour *C_t_*_−1_ (the blue boundary in Figure 3a) of the previous frame *I* at time *t* − 1, we need to get an accurate contour *C_t_* (the blue boundary in Figure 3f) in the current frame *J* at time *t*. The processing method at each frame includes three steps: tracking features, estimating coarse contour and obtaining accurate contour.

### 5.1. Tracking Features

In the feature tracking step, high quality features need to be detected in real-time. The fast corner detection method using machine learning to classify patches of the image as corners or non-corners is used to get good features in the previous frame *I*. It has been shown to be faster than existing feature detectors such as Harris, SUSAN and SIFT (DoG) [26] and outperforms them all in terms of speed. We discard all the features not in the object boundary and only track these *K* remaining features in the current frame *J*. The detected features in previous frame *I* are the green pixels as shown in Figure 3b. Then the Lucas-Kanade optical flow tracker based on image pyramids [27] is used to track the features. For a feature point *u* = (*u_x_*, *u_y_*) on frame *I* at time *t* − 1, the goal is to find its corresponding location *v* = *u* + *d* = (*u_x_* + *d_x_*, *u_y_* + *d_y_*) on frame *J* at time *t*. The vector *d* is the image velocity and minimizes the residual function *ε. ε* is defined as:

(9) $$\varepsilon (d)=\varepsilon (d_{x},d_{y})=\sum_{x=u_{x}-w_{x}}^{u_{x}+w_{x}}\sum_{y=u_{y}-w_{y}}^{u_{y}+w_{y}}{\left(I(x,y)-J(x+d_{x},y+d_{y})\right)}^{2}$$

where *w_x_* and *w_y_* are constant with typical values of 2, 3, 4, 5, 6, 7 pixels. Define spatial gradient matrix:

(10) $$G=\sum_{x=u_{x}-w_{x}}^{u_{x}+w_{x}}\sum_{y=u_{y}-w_{y}}^{u_{y}+w_{y}}\left[\begin{array}{cc} I_{x}^{2}(x,y) & I_{x}(x,y)I_{y}(x,y)\\ I_{x}(x,y)I_{y}(x,y) & I_{y}^{2}(x,y) \end{array}\right]$$

where *I_x_* (*x*,*y*) and *I_y_* (*x*,*y*) are the gradient in the *x* and *y* directions.

Define image mismatch vector:

(11) $$b_{k}=\sum_{x=u_{x}-w_{x}}^{u_{x}+w_{x}}\sum_{y=u_{y}-w_{y}}^{u_{y}+w_{y}}\left[\begin{array}{c} I_{k}I_{x}(x,y)\\ I_{k}I_{y}(x,y) \end{array}\right]$$

where the *k^th^* image difference *I_k_* is defined as follows:

(12) $$\forall (x,y)\in [u_{x}-w_{x},u_{x}+w_{x}]\times [u_{y}-w_{y},u_{y}+w_{y}],\;\;\;\;\;\;\;\;\;I_{k}(x,y)=I(x,y)-J_{k}(x,y)$$

where *J_k_* is the new translated image. Compute the optimal solution of *d* using the Lucas-Kanade optical flow computation:

(13) $$d_{\mathrm{opt}}=G^{-1}b_{k}$$

*d_opt_* is the optimal solution in theory, in practice, we compute it using iterative computations. Suppose the residual optical flow *η_k_* = *G*^−1^*b_k_*, the pixel displacement guess *d_k_* is defined as:

(14) $$\{\begin{array}{c} d_{0}=0\\ d_{k}=d_{k-1}+\eta_{k}(k\geq 1) \end{array}$$

For the pyramid representations of frame *I* and *J*, the image matching error at level *L* is defined as:

(15) $$\varepsilon (d^{L})=\varepsilon (d_{x},d_{y})=\sum_{x=u_{x}-w_{x}}^{u_{x}+w_{x}}\sum_{y=u_{y}-w_{y}}^{u_{y}+w_{y}}{\left(I^{L}(x,y)-J^{L}(x+d_{x}^{L}+g_{x}^{L},y+d_{y}^{L}+g_{y}^{L})\right)}^{2}$$

where *g^L^* is the initial pyramidal guess at level *L* which is available from the computations done from level *L_m_* (the value of *L_m_* is the height of the pyramid) to level *L* + 1. The expression of *g^L^* is

(16) $$\{\begin{array}{c} g^{L_{m}}=0\\ g^{L}=2(g^{L+1}+d^{L+1}) \end{array}$$

The final optical flow solution *d* is then available after the finest optical flow computation:

(17) $$d=g^{0}+d^{0}=\sum_{L=0}^{L_{m}}2^{L}d^{L}$$

Therefore, the corresponding point of feature, point *u*, is found. The tracked features in the current frame *J* are the green pixels as shown in Figure 3c. This tracking method is proven to be robust and all computations are kept at a subpixel accuracy by using bilinear interpolation.

### 5.2. Estimating Coarse Contour

After tracking the features, the coarse contour of the object in the current frame *J* can be estimated. We compute the average displacement *D_t_* of the matched features between previous frame *I* and current frame *J*:

(18) $$D_{t}=\frac{\sum_{(i,j)\in M}f_{i,t}-f_{j,t-1}}{\left|M\right|}$$

where *M* is the set of matched features, *f_i,t_* and *f_j,t−_*_1_ are two matched features in frame *I* and *J* respectively. The coarse contour *Ĉ_t_* of the object in the current frame *J* is simply the previous contour *C_t_* with the foreground pixels translated by *D_t_*. Figure 3d shows the coarse contour of the current frame *J*.

### 5.3. Obtaining Accurate Contour

To get the accurate object boundary, we first dilate the coarse contour *Ĉ_t_* by eight pixels to produce a band area *B_t_*. All pixels in the inner boundary are automatically labeled as foreground pixels and all pixels in the outer boundary are automatically labeled as background pixels. The blue area of Figure 3e is an example of a band. To obtain an accurate contour we use the same method as mentioned in Section 4, which is made up of two steps: hidden information analysis and graph-based image segmentation. By contrast with the method mentioned in Section 4, we don’t construct a graph of the entire image, the graph will only contain nodes for pixels in band *B_t_*. Because *B_t_* typically covers less than 1% of the entire image, the tracking procedure is guaranteed to be real-time.

## 6. Occlusion Handling

**Figure 4.**
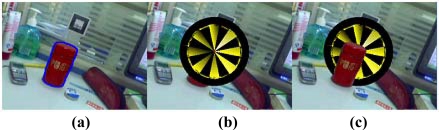
An example of occlusion handling. (a) The tracked contour of the real object. (b) Augmented image with the wrong relationship between the real and virtual objects. (c) Synthesized image with the correct relative relationship.

This section deals with the actual mechanics of the occlusion handling. Suppose the intrinsic camera parameters are known in advance and do not change. First, we need to get the current frame with the virtual object rendered in it. We use the standard marker based approach to render the virtual objects. This requires three steps: (1) search for the marker in the current frame; (2) calculate the position and orientation of the marker; (3) align the 3D virtual object with the marker by transforming them using the position and orientation parameters calculated in step 2. The result is that we get the augmented image frame with the virtual object overlaid on the marker. In our implementation, this is done using ARToolKit [28]. The augmented image will have the wrong occlusion relationship between real and virtual objects when the real object occludes the virtual object. The next step is to acquire the correct relationship between real and virtual objects. We redraw all the pixels on the physical tracked object in a new synthesized image. This simple process can effectively deal with the occlusion problem in augmented reality. Moreover, the border between the virtual and the occluding real object is smoothed to be seamless. An example of the occlusion handling process is shown in Figure 4.

## 7. Experimental Results

The proposed approach has been implemented using Visual C++, OpenCV [29] and ARToolKit on a 1.9 GHz CPU with 512 MB RAM. The video sequences are captured using a Logitech Pro5000 camera. In all cases, the input images had a resolution of 320 × 240 pixels. The camera’s intrinsic parameters are solved in advance using the method introduced in [30]. The system can run the proposed approach at a speed of about 18 frames per second. Several experiments have been conducted to test the proposed occlusion handling method.

**Figure 5.**
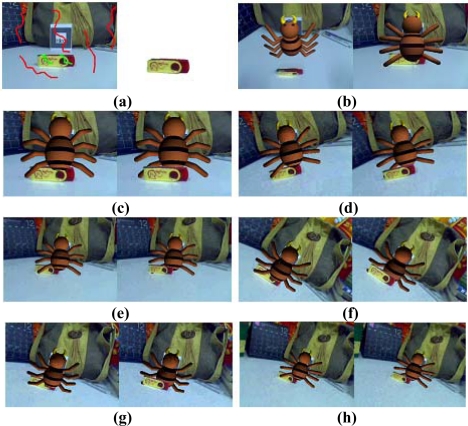
Results of the first experiment to test the proposed occlusion handling method.

In the first experiment, the user labels a few pixels with green lines to represent the foreground and a few pixels with red lines to represent the background in the first frame (Figure 5a). The physical object is then segmented from the background using the segmentation method based on mean shift and graph cuts. The physical object is tracked using the method combining optical flow and graph cuts in the subsequent frames. Finally, we redraw all the pixels on the tracked physical object of the augmented image to produce a new synthesized image with the correct relative relationship. A smoothing process is undertaken to make the boundary between the physical and virtual object seamless. In this experiment, the virtual model is a spider. The left image of Figure 5b shows that the USB stick is in front of the virtual spider, so that if the user has a viewpoint as shown in the right image of Figure 5b, part of the virtual spider should be occluded by the USB stick. The right image of Figure 5b shows the incorrect relative relationship between the physical and virtual objects without occlusion handling. Figure 5c–h shows the comparison between the incorrect occlusion images shown in the left and the correct occlusion images shown in the right. These results demonstrate that our approach can effectively handle the occlusion problem.

**Figure 6.**
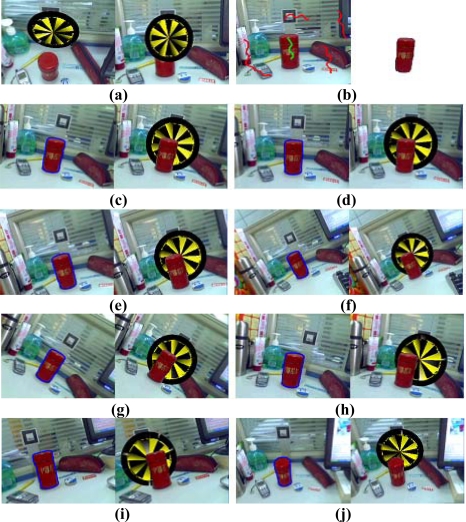
Results of the second experiment.

The segmentation result in the first frame may not be satisfied because of insufficient user interaction, but this does not harm the results of the occlusion handling. The physical object boundary computed in the first frame need only be approximate, because the segmentation boundary will generally converge onto the object boundary within a few frames. As an example, in the second experiment, as shown in Figure 6a, there are two augmented images seen from different viewpoint. Figure 6b shows that the boundary of the segmented tea box in the first frame is blurry. Many foreground pixels around the object boundary are misclassified as background pixels. However, after a few frames, the computed object boundary converges onto the true object boundary and the edge is clear. Figure 6c–j demonstrates that the results of the occlusion handling are robust even the boundary in the first frame is inaccurate and the viewpoint of the camera changes.

**Figure 7.**
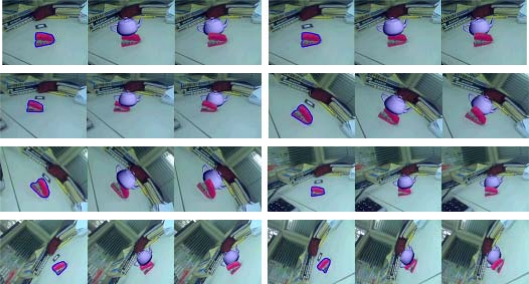
Results of the third experiment.

When the pixels on the object are similar to the background, it is difficult to track the object in the subsequent frames. This problem may lead to incorrect results in the occlusion handling. In the third experiment, the virtual model is a teapot. Some pixels of the stapler have almost the same color as the desktop. The results shown in Figure 7 demonstrate that our approach is effective even in the difficult case when the camera changes over a wide range of viewing angles and volumes.

To show that our approach can meet the real-time requirements, the average processing time of each step are shown in Table 1. The step of selecting the physical object is implemented only in the first frame. Thus the total processing time at each frame is the sum of occluding physical object tracking time and occlusion handling time which takes about 0.053 second per frame on the average. This processing time satisfies the real-time requirement in the augmented reality systems.

## 8. Discussion

Our experimental results demonstrate that our algorithm can successfully and robustly handle the occlusion problem for augmented reality applications in real-time. The total running time is proportional to the size of the object and the size of the video image captured. However, our approach is still performs well even when the object covers a large area of the image or when the video image is large. For an input image with a resolution of 640 × 480 pixels, the average processing time is about 10 frames per second.

However, there are still some disadvantages of our method. After the user specifies the occluding object, the object will occlude the virtual object all the time. With the camera moving all about the virtual object, our approach will lead to incorrect occlusion relationships when the virtual object is between the camera and physical object. In other words, the camera can be moved around the virtual object in a limited angle, from 0 to 180 degrees.

Another limitation of our algorithm will occur if the virtual object is surrounded by a large real object and the virtual object is occluded by some real parts and then occludes other real parts. In such a situation our method will fail, meaning that our method can solve the occlusion problem when the virtual object is behind the real object, but not when the virtual object is surrounded by a large real object.

Based on the above discussions, our method is good for desktop systems using a camera moving around the virtual object with a limited angle (from 0–180 degrees) and not systems using a handheld or head-mounted display with lots of camera movement. To overcome the problems mentioned above, we consider modeling the whole scene to get the depth map of the scene from different angles of view. In this way, the depth of each pixel in the image captured from any viewpoint is known, and then the correct occlusion relationship can be obtained by comparing the depth.

## 9. Conclusions and Future Work

In this paper, we have proposed an effective real time occlusion handling method based on an object tracking approach. Correct relative positions between real and virtual objects can be obtained using our approach. Experimental results show that the proposed method is effective, robust and stable, even in the case of a poor initial object boundary. The approach can deal with complex scenes and large changes in viewpoints and volumes. Moreover, our method can operate in real-time. However, when the camera moves too fast, the tracking process may fail and we do not get the correct occlusion handling results. In the future, we will work on some methods to deal with this problem. Furthermore, in AR systems, the ideal occlusion handling method is to automatically handle the occlusion problem without user interaction. In future work, we will consider automatically detecting the occlusion relationship between the real and virtual objects using depth map. By comparing the depth of each pixel on the real and virtual objects, the correct occlusion relationship can be obtained without user interaction. This approach will reduce the manual operation and this is the research emphasis of our future work.

## Figures and Tables

**Table 1.** Processing time of each step.

Steps		Average processing time (seconds)	Total time (seconds)
Occluding real object specifying	hidden information analysis	0.153	0.231
	graph-based image segmentation	0.078	
Occluding real object tracking	feature tracking	0.007	0.053
	coarse contour estimating	0.000	
	accurate contour obtaining	0.031	
Occlusion handling		0.015	
